# Health problem behaviors in Iranian adolescents: a study of cross-cultural adaptation, reliability, and validity

**Published:** 2010

**Authors:** Ahmad Ali Eslami, Fazlollah Ghofranipour, Bagher Ghobari Bonab, Davood Shojaei Zadeh, Farkhondeh Amin Shokravi, Mahmoud Ghazi Tabatabaie

**Affiliations:** a Student of Health Education, Faculty of Medical Sciences, Tarbiat Modares University, Tehran, Iran; b Department of Health Education, School of Health, Isfahan University of Medical Sciences, Isfahan, Iran; c Department of Health Education, Faculty of Medical Sciences, Tarbiat Modares University, Tehran, Iran; d Department of Special Education, Psychology and Education University of Tehran, Tehran, Iran; e Department of Health Education, Faculty of Health Sciences, Tehran University of Medical Sciences, Tehran, Iran; f Department of Health Education, Faculty of Medical Sciences, Tarbiat Modares University, Tehran, Iran; g Department of Demography, Faculty of Social Sciences, Tehran University, Tehran, Iran

**Keywords:** Adolescence, Problem Behavior Syndrome, Low Self Concept, Emotional Distress

## Abstract

**BACKGROUND:**

The main purpose of this study was to assess the factorial validity and reliability of the Iranian versions of the personality and behavior system scales (49 items) of the AHDQ (The Adolescent Health and Development Questionnaire) and interrelations among them based on Jessor’s PBT (Problem Behavior Theory).

**METHODS:**

A multi-staged approach was employed. The cross-cultural adaptation was performed according to the internationally recommended methodology, using the following guidelines: translation, back-translation, revision by a committee, and pretest. After modifying and identifying of the best items, a cross-sectional study was conducted to assess the psychometric properties of Persian version using calibration and validation samples of adolescents. Also 113 of them completed it again two weeks later for stability.

**RESULTS:**

The findings of the exploratory factor analysis suggested that the 7-factor solution with low self concept, emotional distress, general delinquency, cigarette, hookah, alcohol, and hard drugs use provided a better fitting model. The α range for these identified factors was 0.69 to 0.94, the ICC range was 0.73 to 0.93, and there was a significant difference in mean scores for these instruments in compare between the male normative and detention adolescents. The first and second-order measurement models testing found good model fit for the 7-factor model.

**CONCLUSIONS:**

Factor analyses provided support of existence internalizing and externalizing problem behavior syndrome. With those qualifications, this model can be applied for studies among Persian adolescents.

Adolescence is described by period of multiple transitions that involves pubertal, relationship, academic, and ability changes^1^ which may contribute to problem behaviors at this period.^2^ More than 60% of children get involved in some kind of problem behaviors in the course of adolescence.^3^ In fact, problem behavior or deviant behavior could be socially defined as problem, a source of concern, a result of a lack of conformity, and its occurrence usually elicit some kind of social control response.^4^ These individual problems may result from general patterns of difficulties in social development.^5^ However, other studies suggest that multiple factors may be needed to explain the interrelations among various problem behaviors among youth.^6^,^7^ Based on these studies, the notion that different problem behaviors reflect a single common factor remains unsupported. A study showed that a single-common factor accounted for the significant interrelations among the different problem behaviors for the entire sample.^8^ The studies indicated the high rates of substance use in Iran that is increasing continually.^9^ For example, a study was found that, majority of adolescents have used one of these drugs at least one time: cigarette (42.3%), alcohol (37.5%), hashish (4.4%), and 4.1% for opium^10^ which has been shown to be a risk factor for coronary artery disease.^11^ Hookah use and its popularities is increasing in Iranian youth.^12^ For example, a study on the cardiovascular risk factors in a sample of adolescent students in Tehran showed that 55% of students (63% of boys and 47% of girls) had experienced hookah smoking.^13^ Now, health problem behavior of adolescents is a main concern for families, society and policy makers. Jessor’s PBT (1977) is a psychosocial model that attempts to explain behavioral outcomes such as substance use, deviancy, and risky sexual behaviors.^8^ Despite empirical support of PBT for explaining interrelations among adolescents’ risk behaviors,^14^ few studies have tested the applicability and generalizability of this model to other countries.^15^,^16^

PBT was developed to explain causes of incidence of behaviors that aren’t socially desired for adolescents. PBT asserts susceptibility to problem behaviors results from the interaction of the person and the environment. Jessor’s theory represents an ecological framework that posits non-conventionality in values, the personality, and the social environment is related to multiple problem behaviors.^14^ Both theory and numerous studies explain the relationships among adolescent high-risk behaviors such as delinquency, drinking, and drug use and the existence of a single “Problem Behavior Syndrome (PBS)” which is generalizable to different gender, socioeconomic and ethnic groups.^17^ In present study low self esteem, low expectations for success as LSC (Low Self Concept), depression and felt stress is conceptualized as ED (Emotional Distress). LSC means that there is a lack of aspects of self such as self-esteem and self worth that are important for a positive outlook on life by adolescents,^18^ and it is an important cause of several behavioral outcomes such as violent and aggressive behavior, depression, substance abuse, and achievement difficulties.^19^ ED consists of behaviors that encompass negative affectivity or the combination of anxiety and depressive features that are central to adolescents who feel greatly distressed about their life.^18^ An empirical study confirmed that higher felt stress is associated with lower self esteem.^20^ Studies have shown that negative affectivity is both predisposing and predictor for involvement in problem behaviors.^21^,^22^ There are studies that has indicated a relation between poor psycho-social adjustment and various internalizing and externalizing problems like ED, suicide ideation, and violence.^23^ It is clear that existence of reliable and valid measures are necessary to apply this theory in studies among Iranian teenagers. Thus, the present study is focused to culturally adapt the variables of the personality and behavior systems of Jessor’s theory to the Farsi language, assessing validity and reliability of its Iranian version, were investigated interrelationships among problem behaviors and the relations between problem behaviors and the personality factors. In spite of the fact that PBT was confirmed on one-factor structure for problem behavior, we also examined the structure of externalizing and internalizing problem behavior among adolescence. The current study has considered LSC and ED as internalizing behaviors that associated with externalizing behavior factors including general delinquency (GD), cigarette, hookah, alcohol, hashish, and opium use.

## Methods

### 

#### Sampling Procedures

A cross-sectional self-report survey method was used in this study. The sample was drawn from all governmental and semi governmental schools of Tehran suburban areas. A stratified, two-stage, cluster sampling procedure were used to make them representative of distribution of demographic characteristics. Participants were stratified into boys and girls. A total of 18 schools were selected using a probability proportional to size (PPS) method for gender stratum (10 male schools and 8 female schools). In the first stage, within each strata, 13 of 57 general high schools (7 for boys and 3 for girls), and 5 of 24 technical/vocational high schools (3 for boys and 2 for girls) were randomly selected. In the second stage, one class from each grade within the sampled schools was randomly determined. Participation of students in this study was voluntary and all information was collected anonymously. Students completed the self-administered questionnaire during a regular class period (40-50 minutes), recording their responses directly on the survey. Signed parental consent forms were required to participate in the study.

#### Instruments

Instruments consisted of 4 subscales of personality system (24 items), and 7 subscales of behavior system (25 items) of the AHDQ (The Adolescent Health and Development Questionnaire). Its English version has been well investigated and its scales psychometric properties are good.^15^,^24^,^25^ In addition, 3 items assigned for assessing the hookah smoking. In this questionnaire, felt stress (α range: 0.68-0.74), depression (α range: 0.78-0.85), low self esteem (α range: 0.68-0.68), low expectations for success (α range: 0.88-0.89), GD (α range: 0.82-0.84), variables were measured with 3, 4, 8, 9, and 10 items respectively. Also, 4 or 5 Likert style was used in all internalizing behavioral scales. Each of 5 subscales, cigarette, hookah, alcohol, hashish, and opium use (α range: 0.79-0.90, 4 or 8 Likert style), consisted of 3 items that adapted from the AHDQ.

#### Data Analysis

According to the established guidelines for self-assessment instruments,^26^ procedures of cross-cultural adaptation were performed: 1) translation/back-translation process and discussion on each item by the research group to achieve conceptual equivalence; and 2) to finalize the adaptation, item clarity, content validity, and length of the questionnaire, a pilot test was carried out with the translated and revised instruments that consisted of 2 steps: (a) instruments were submitted by 7 academic experts who were specialist in the measures and concepts involved. The experts rated relevance of each question on a Likert type ordinal scale (1 = not relevant to 4 = very relevant). (b) Cognitive debriefing was performed with 34 students of the target population to assess respondent comprehension and the feasibility of the instruments. Participants rated the difficulty they experienced in answering and understanding the items on a 4-point categorical scale (1 = no difficulty to 4 = severe difficulty). For goal two, a two-step SEM approach was used. Preliminary analysis was performed on the calibration sample to establish factorial validity, and then cross-validated on a validation sample. In the first stage, principal components analysis (PCA) using non-orthogonal promax rotation procedure (K = 4) conducted. The factor structure was assessed using several criteria, including (a) analysis of the Eigen values greater than 1 in the Scree plot, (b) item cutoff loading greater than or equal to 0.40, and (c) meaningfulness of the observed factors.^27^ This method has also been used in other fields.^28^ Internal consistency was assessed means of Cronbach α for the items of the each scale. The stability was evaluated using the intra-class correlation coefficient (ICC) from a two-way random effects model, single measure (2, 1) and an ICC value of at least 0.70.^29^ The Mann-Whitney U test was used to asses differences in scores of instruments between the male students and detente adolescents (because of involvement in delinquency and substance use). The measurement models were assessed on the calibration to establish factorial validity, and then cross-validated on a validation sample by the program LISREL (8.8). Because of some items exhibited severely high estimates of non-normality, the confirmatory factor analysis using un-weighted least squares (ULS) estimation method was selected.^30^

## Results

### 

#### Demographic Data

The participants consisted of 1003 (608 male, 395 female) students drawn from 54 classrooms in 18 high schools of Tehran suburban area. Of the 1003 selected students, 91% (912/1003) students agreed to participate in the survey; 3% (27/912) students were excluded from analyses resulting because of substantial missing data, i.e. problem behaviors. The final sample included 885 high school students (Mean ± SD of age = 16.7 ± 0.74 years, age range : 15 to 19) that were in the 9th (27.5%), 10th (36%), or 11th (36.5%) grades. In addition, 102 boy-eleventh grade- (102/115) completed the questionnaire two weeks later. This subsample’s age ranged from 17 to 19 years (Mean ± SD = 17.80 ± 0.59). SES was estimated based on the father’s job, family income, and perceived family welfare. There were no significant group differences on these variables.

#### Cultural Adaptation Process

To achieve semantic equivalence, procedures of translation, and back-translation were performed. The results did not showed main differences to alter the meaning of the questions or to remove. Some words and sentences modified to adapt them to Farsi language and culture. The mean CVI was 0.89 (r = 0.57-1); that relatively was high. The questionnaire was piloted among a group of 34 high school students. Based on the pilot test findings, 6 items were added to the section of problem behavior about traditional drugs, such as hookah and opium.

#### The Exploratory Factor Analysis, Homogeneity, and Stability

All of the items were entered into the PCA. The PCA resulted in a 9-factor solution with Eigen-values > 1, and criterion > 0.40. This factor structure explained 61.34% of total variance. The Scree plot test indicated to flatten after 7-factor. Thus, 7-factor solution was retained and was conceptually meaningful, and nearly to that reported by Jessor et al, (2003).^15^ This structure accounted for 56.14% of the total variance (see Appendix A). Based on the exclusion criteria presented above, items 3, 16, 25, 28, 44 and 47 were excluded from the next analysis. The first component explained 35.4% of the variance, and was conceptualized as hard drugs use (HDU) (α = 0.94). Second component explained 6.9 % of the variance, and was conceptualized as LSC (α = 0.88). The third component was original GD subscale (α = 0.87), that explained 5.3% of the variance. The forth component explained 3.6% of the variance, and was conceptualized as ED (α = 0.71). The components of 5-7, all together, explained 8.5% of the variance (hookah (α = 0.90), alcohol (α = 0.92), and cigarette (α = 0.92) use subscales). Indeed, CFA with Eigen value > 1.5 clearly indicated a 4-factor solution that included substance use, GD, ED, and LSC. Alpha coefficients ranged from 0.71 to 0.94 that indicated excellent internal reliability. ICC (2, 1) values with a 2-week interval (n = 113) showed good temporal stability for all sub-scales (ICC range: from 0.73 to 0.93). As expected, Mann-Whitney test showed a significant difference in mean scores for these instruments (Table 1).

**Table 1:** Internal consistency, test-retest reliability, and known-group validity

Components (n of items)	α (n = 430)	ICC* (n = 113)	Group 1 (n = 97)	Group 2 (n = 35)	P**
			Mean ± SD	Mean ± SD	
Hard drugs use (4)	0.94	0.93	7.1 ± 3.6	10.9 ± 6.9	< 0.001
Low self concept (15)	0.88	0.81	37.9 ± 6.0	41.2 ± 4.5	< 0.01
Delinquency (8)	0.87	0.83	20.1 ± 4.7	27.7 ± 5.4	< 0.01
Emotional distress (7)	0.71	0.73	15.0 ± 3.3	18.0 ± 3.2	< 0.001
Hookah use (3)	0.90	0.87	5.7 ± 3.6	10.9 ± 3.6	< 0.001
Alcohol use (3)	0.92	0.87	4.5 ± 4.1	7.6 ± 4.0	< 0.001
Cigarette use (3)	0.92	0.90	4.8 ± 3.2	8.6 ± 4.6	< 0.01

* Intra-class correlation coefficient

** Mann-Whitney U-test

#### Model Testing

Models were compared using multiple fit indices that was recommend by Hu et al (1999).^31^ The fit indices used were the Satorra-Bentler chi-square (S-B χ2) that serves as a correction for the χ2 statistic when the distributional assumptions are violated; the non-normed fit index (NNFI), the comparative fit index (CFI), and the root mean square error of approximation (RMSEA). Table 2 shows the zero-order correlation matrix, with means and standard deviations of variables that identified in the CPA, separated by both samples. As observed, correlations between scores are moderate to high.

**Table 2:** Correlations matrix, means, and standard deviations for study variables across samples of calibration (n = 430) and validation (n = 455)

Components		1	2	3	4	5	6	7
Hard drugs use	1	1	0.73 *	0.467 *	0.71 *	0.52 *	0.70 *	0.62 *
Cigarette use	2	0.67 *	1	0.373 *	0.36 *	0.46 *	0.64 *	0.57 *
Alcohol use	3	0.49 *	0.40 *	1	0.24 *	0.38 *	0.50 *	0.41 *
Hookah use	4	0.36 *	0.46 *	0.377 *	1	0.56 *	0.55 *	0.44 *
Delinquency	5	0.58 *	0.59 *	0.541 *	0.60 *	1	0.53 *	0.62 *
Emotional distress	6	0.35 *	0.46 *	0.329 *	0.24 *	0.45 *	1	0.54 *
Low self concept	7	0.36 *	0.49 *	0.230 *	0.250 *	0.57 *	0.511 *	1
Mean ± SD	Calibration	6.7 ± 2.8	4.7 ± 3.0	4.0 ± 2.9	5.7 ± 3.6	17.5 ±5.0	15.4 ±3.3	35.0 ± 5.6
	Validation	6.4 ± 1.9	4.3 ± 2.7	3.8 ± 2.5	5.4 ± 3.4	16.9 ± 4.3	15.4 ± 3.1	34.9 ± 5.1

Correlations for validation sample shown above and for calibration sample shown below the diagonal.

* p < 0.01

The first-order measurement models include: a) the 7-factor solution with 43 manifest indicators that identified in the PCA on the calibration sample, and b) the 4-factor solution (substance use, GD, ED, and LSC) that were extracted with Eigen value greater than 1.5. Because of the high skewness and kurtosis values of some of the drug use indicators, the parcel method was applied. Thus, to compare, more parsimonious 7- and 4-factor solutions corresponding models based on item parcel were created (Appendix B–C). Each of the item parcels were arbitrarily formed by calculating average items that were adjacent and conceptually similar.^32^ For model identification, at least three manifests were used for each sub-scale.^33^ The results of CFAs for all models are shown in table 3. The findings showed that the best fitting model was model 1. Fit indices for 7-factor models are all within good range according to Hu et al (1999) criteria. The 7-factor models (models 1 and 2) provide evidence of consistent of fit indices across two samples. Model 1 fit indices (with 43 indicators, and 7-factor) showed fits the data relatively good and consisted between two sample (S-Bχ2 (839) = 1203.5, CFA and NNFI = 0.99, PNFI = 0.98, RMSEA = 0.033, for calibration sample; S-Bχ2 (839) = 1175.9, CFA = 0.99, NNFI = 0.98, PNFI = 0.088, RMSEA = 0.032, for validation sample).

**Table 3:** Goodness-of-fit indices for models 1 to 6 across calibration and validation samples

Models	Description	SB-χ2	df	CFI	NNFI	PNFI	RMSEA
Calibration							
Ml (IB)	7 F (43 I)	1203.5	839	0.99	0.98	0.90	0.033
M2 (PB)	7 F (24 I)	0405.3	231	0.99	0.99	0.82	0.042
M3 (IB)	4 F (43 I)	3657.3	854	0.93	0.92	0.85	0.087
M4 (PB)	4 F (24 I)	2273.2	246	0.91	0.90	0.80	0.130
M5 (PB)	7 F, two second-order factors	0487.0	244	0.99	0.99	0.87	0.048
Validation							
Ml (IB)	7 F (43 I)	1175.9	839	0.99	0.98	0.88	0.032
M2 (PB)	7 F (24 I)	0350.6	231	0.99	0.99	0.82	0.034
M3 (IB)	4 F (43 I)	4116.8	854	0.89	0.88	0.82	0.092
M4 (PB)	4 F (24 I)	2121.0	246	0.90	0.88	0.79	0.110
M5 (PB)	7 F, two second-order factors	393.4	244	0.99	0.99	0.86	0.037
Full sample							
M6 (PB)	7 F, one second-order factors	0767.1	243	0.99	0.99	0.86	0.049

SB-χ2 = Satorra-Bentler chi-square; df = Degrees of freedom; CFI = Comparative fit index; NNFI = Non-normed fit index; PNFI = Parsimony normed fit; RMSEA = Root mean square error of approximation; M = Model; F = Factor; I = Indicator; IB = Item based; PB = Parcel based

To compare the 7-factor solutions and considering CFA results a 4-factor solution corresponding model was built. To test the 4-factor solution substance use, items of cigarette, hookah, alcohol, hashish, and opium use were assigned to one single factor as was also done in the similar studies.^34^,^35^ Goodness-of-fit indexes for 4-factor model (model 3) did not show acceptable fits (e.g., RMSEA values for model 3 were 0.087 and 0.092 across two samples). Therefore, alternative model 3 was rejected (Table 3).

**Figure 1:**
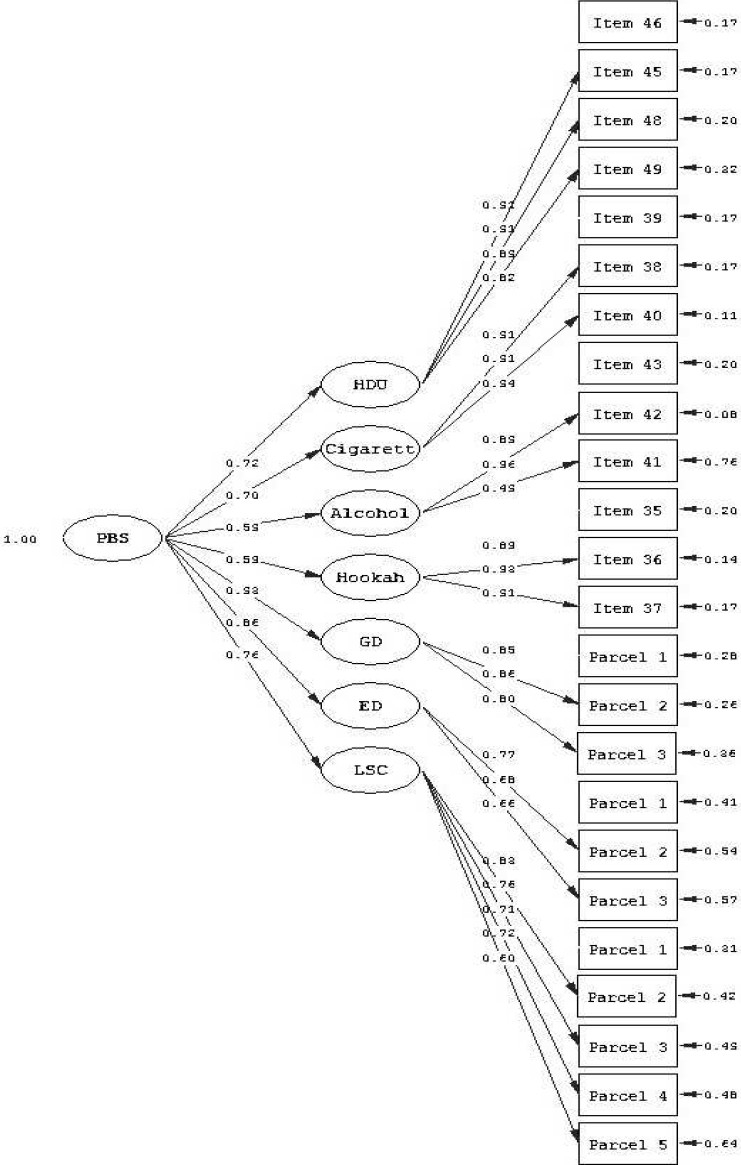
Final one factor second order measurement model for problem behavior syndrome PBS = Problem behavior syndrome; HDU = Hard drugs use; GD = General delinquency; ED = Emotional distress; LSC = Low self concept

Also two models were included based on parcel in analysis. findings showed that model 2 with 24 manifest indicators and 7 first order latent factors also indicated a good fit across two samples (S-Bχ2 (231) = 405.3, CFA and NNFI = 0.99, PNFI = 0.98, RMSEA = 0.042, for calibration sample; S-Bχ2 (231) = 350.6, CFA = 0.99, NNFI = 0.99, PNFI = 0.082, RMSEA = 0.034, for validation sample). Model 4 was consisted of 24 manifest indicators and 4 first order latent factors. In model 4, scales of substance use applied as 3-4 items parcels. The model fit also was poor among two samples (SBχ2 (246) = 2273.2, CFA = 0.91, NNFI = 0.90, PNFI = 0.80, RMSEA = 0.13, for calibration sample; S-Bχ2 (246) = 2121.0, CFA = 0.90, NNFI = 0.88, PNFI = 0.79, RMSEA = 0.11, for validation sample). In examining the model fit indices, 7-factor models had RMSEA indices in the good range. However, between model 1 and 2, model 1 was not selected in subsequent analysis because it was a less parsimonious model (with one path more) than model 2. A two-factor second-order model was created on the basis of the results of a series of first-order factor analysis. Model 5 included 24 indicators, 7 first order latent components (LSC, ED, GD and 4 substance use scales) and two second-order latent factors that representing the internalizing and externalizing problem behavior constructs. Results of the tests of the second-order model of problem behaviors across two samples are presented in table 3. Model 5 showed the highest association (r = 0.87) between externalizing and internalizing problem behaviors in studying sample. Fit statistics indicated that two-factor second-order model provided a good fit (S-Bχ2 (244) = 487.0, CFA = 0.99, NNFI = 0.99, PNFI = 0.87, RMSEA = 0.048, for calibration sample; S-Bχ2 (244) = 208.6, CFA = 0.99, NNFI = 0.99, PNFI = 0.86, RMSEA = 0.037, for validation sample). The high correlation and the previous findings showed existence of the one-factor second-order model and PBS. Therefore, a final one-factor second-order model (model 6) runs over whole sample. Results did not indicated a main difference in model with two-factor second-order model (S-Bχ2 (243) = 767.1, CFA = 0.99, NNFI = 0.99, PNFI = 0.86, RMSEA = 0.049). All first and second-order factor loadings were high (Figure 1). The results of the goodness-of-fit indices for the model 6 over full sample are presented in table 3.

## Discussion

This study reports on the results of the cultural adaptation, feasibility, factorial validity and reliability of the personality and behavior factors (49 items) of the AHDQ that its content theoretically derived from the constructs in Jessor’s PBT, following international methodological procedures and examining the role of personality variables as internalizing problem behavior. Reliability and validity of instruments in this study were carried out through a rigorous process of an initial pilot study, translation, back-translation, cultural adaptation, and, finally, a validation study. As a result, the psychometric properties of obtained Iranian version instruments in the present study relatively are closed to original versions. It was found that 43 of the 49 items loaded into 7-factor in PCA that accounted for 56.14% of the total variance. Although there was substantial overlap in scale content, some of the items loaded on to different factors. However, some of the items may not completely reflect the cultural characteristics or connotative meaning of the English version. For example, the items such as “to cheat on tests or homework” and “to lie to a teacher” had factor loadings under 0.30. Internal consistency instruments showed acceptable standards for reliability (α range: 0.71-0.94).The stability of each of the 7-factor over time was assessed by examining over a 2-week interval the ICC (range: 0.73 for ED to 0.93 for HDU) that is higher than the recommended value of 0.70.^29^ As findings showed, there was a significant difference in mean scores for the present instruments among two groups (high school’s and detention’s male samples). It means that the instruments allow discrimination between adolescents with different levels of problem behavior severity. However, the instruments used in general population,^15^,^24^,^25^ thus it was recommended that obtained Iranian versions in the present study were used for in general population. This study also examined the interrelationships among measures of problem behaviors in calibration and validation samples. Measures of these problem behaviors were positively interrelated. Correlations between factors were similar to those reported in other studies.^36^ As expected, low self concept as a proximal factor was correlated to adolescents’ reports of both internalizing and externalizing problems. This finding is consistent with results of some other studies.^19^ For example, the low self concept had high correlation with delinquency, emotional distress, smoking, alcohol and drugs use.^20^ In some studies emotional distress was correlated with externalizing problem behaviors.^21^ In addition, the present study showed a high positive correlation between delinquency and drugs use. These results are in line with previous studies.^37^ Because of high reports on hookah use in Iranian youth,^13^,^38^ it was necessary to examine relationships between hookah use and other problem behaviors. The present study provided strong support for the hypothesis that hookah smoking is a part of the problem behavior syndrome Figure 1. Next, four first order measurement models and one-second-order model with two higher-order factor were tested across calibration and validation samples. This method is required to make appropriate group comparisons.^39^ Main systematic variation didn’t exist in relation to major demographic categories within the sample. The results of the confirmatory factor analyses demonstrated the adequacy of the first-and second-order CFA models (identified in PCA analysis) of PBS in data of two samples. Multi-sample analyses indicate that the 7-factor first and second-order models of PBS satisfactorily described the data across the two national samples. Results obtained from the first-order CFA models supported 7-factor structure that extracted from PCA. In contrast, 4-factor models that were derived from the present interpretation of the extant theoretical and meta-evaluation evidence pertinent to the conceptualization of substance use disorder,^22^ indicated a poor model fit. In spite of the high adequacy of the 7-factor first-order model, a higher-order model with two second-order latent factor was estimated. Although several studies were examined PBS structure,^7^ limited empirical evidence exists for the 2-factor second-order model. These two factors therefore labeled as internalizing and externalizing. Findings suggested that second-order model has provided influential insight. Fit statistics indicated that Health problem behaviors in adolescents Eslami et al two-factor second-order model across samples provided a good fit. The correlation between the second-order Internalizing and externalizing factors was 0.87. This high correlation was symptom of existence of the one-factor second-order model. A final one-factor second-order model was calculated.

## Conclusions

In conclusion, in spite of the fact that parcels were used in the CFA, model 6 exhibited a good fit to the sample data. Finally, this study will enhance our understanding of the Iranian adolescents’ behavior particulars. Also, the present research offers a measurable 7-factor model that consists of PBS-related key constructs in study population.

### 

#### Limitations and Implications

Overall, results of present research can help researchers in comparative and preventive studies among adolescent groups. An important precaution to the present findings is about interpretation and generalizability of the results, because problem behavior structure is complex and has an ecological feature. Data are related to the restricted range of adolescents from four areas in Tehran suburban. A worthy result of this research is offering an empirical framework for more other studies. So, obtained problem behavior model and instruments of this study are valid and reliable that can be used in assessing of internalizing and externalizing problem behaviors in the school context.

## Appendix

**Table:** Appendix A. Eigen values, variance and factor loadings explained for the 7-factor derived from principal components analysis of items

Components and items	FL	Components and items	FL	Components and items	FL
LSC (15) (EV = 3.1%, V = 6.9)		ED (7) (EV = 1.6%, V = 3.6)		GD (8) (EV = 2.4%, V = 5.3)
6	0.64	19	0.70	34	0.78
15	0.61	20	0.60	31	0.73
4	0.61	21	0.59	29	0.73
13	0.60	23	0.57	30	0.72
9	0.59	22	0.56	26	0.71
5	0.58	18	0.55	27	0.70
12	0.58	24	0.52	33	0.70
10	0.58	Hard drugs (6)		32	0.68
8	0.57	(EV = 15.9%, V = 35.4)		26	0.71
17	0.56	48	0.94	Alcohol use (3)	
14	0.54	45	0.93	(EV = 1.3%, V = 2.8)	
1	0.54	46	0.92	42	0.98
11	0.54	49	0.89	41	0.96
7	0.53	Cigarette use (3)		43	0.95
2	0.52	(EV = 1.0%, V = 2.3)		Hookah use (3)	
		39	0.92	(EV = 1.5%, V = 3.4)	
		38	0.90	36	0.96
		40	0.85	35	0.94
				37	0.91
Items loading more than 0.4 are reported; FL = Factor loadings; EV = Eigen value; V = Variance; PBS = Problem behavior syndrome; HDU = Hard drugs use; GD = General delinquency; ED = Emotional distress; LSC = Low self concept					

## Appendix

**Table:** Appendix B. Operationalization of measures and constructs of problem behavior syndrome (PBS); (Reference: Jessor et al (2003))

Low self esteem (LSE); 8 Items:	
LSE 1	How well do you get along with others your age?
LSE 2	What about your ability to do well in school work?
LSE 3	How do you feel about the way you look?
LSE 4	How well are you able to handle setbacks and disappointments?
LSE 5	How well do you make decisions about important things in your life?
LSE 6	How much common sense do you have for dealing with everyday problems?
LSE 7	How physically attractive do you think you are to other people?
LSE 8	On the whole, how satisfied are you with yourself?
Low expectations success (LES); 9 Items:	
Think about how you see your future. What are the Chances that:	
LES 9	You will graduate from high school?
LES 10	You will have a job that pays well?
LES 11	You will be doing the kind of work that you like?
LES 12	You will have a happy family life?
LES 13	You will be respected by other people?
Think about how you are doing in school	
LES 14	Get at least a B average this year?
LES 15	Be considered a bright student by your teachers?
LES 16	Come out near the top of the class on exams?
LES 17	Have good enough grades to get into college?
Item Parcels	Parcel 1 = Items 4, 5, 6, and 8; Parcel 2 = Items 1, 2, and 7; Parcel 3 = Items 9, 10, and 13; Parcel 4 = Items 11 and 12; Parcel 5 = Items 14, 15, and 17
Felt stress (FS)-3 Items,- In the past six months, how much stress or pressure have you felt, like not being able to meet all, the demands on you or not being able to get everything done that you need to:	
FS 18	At school?
FS 19	At home?
FS 20	In your personal or social life?
Perceived depression (PD)-4 Items-In the past six months, have you	
PD 21	Just felt really down about things?
PD 22	Felt pretty hopeless about the future?
PD 23	Just felt depressed about life in general?
PD 24	Thought seriously about suicide or ending your life?
Item Parcels	Parcel 1 = Items 18, 19, and 20; Parcel 2 = Items 21 and 22; Parcel 3 = Items 23 and 24
General delinquency (GD)-10 Items-During the past six months, how often have you:	
GD 25	Cheated on tests or homework?
GD 26	Shoplifted from a store?
GD 27	Damaged or marked up public or private property on purpose?
GD 28	Lied to a teacher about something you did?
GD 29	Taken something of value that doesn’t belong to you?
GD 30	Stayed out all night without permission?
GD 31	Lied to your parents about where you have been or who you were with?
GD 32	Hit another student because you didn’t like what he or she did?
GD 33	Carried a weapon, like a knife or gun, at school?
GD 34	Made fun of or picked on other kids because theyare different or not part of your group?
Item Parcels	Parcel 1 = Items 26, 30, 31, and 8; Parcel 2 = Items 27, and 29; Parcel 3 = Items 32, 33 and 34
